# Predicting the spatio-temporal distribution of *Culicoides imicola* in Sardinia using a discrete-time population model

**DOI:** 10.1186/1756-3305-5-270

**Published:** 2012-11-22

**Authors:** Thibaud Rigot, Annamaria Conte, Maria Goffredo, Els Ducheyne, Guy Hendrickx, Marius Gilbert

**Affiliations:** 1 Biological control and spatial ecology (LUBIES), Université Libre de Bruxelles, Av F.D. Roosevelt 50, Brussels, B-1050, Belgium; 2 INRA, UMR 1202, Biodiversity Genes and Communities, Cestas, F-33610, France; 3 Istituto Zooprofilattico Sperimentale dell’Abruzzo e del Molise ‘G. Caporale’, Via Campo Boario, Teramo, 64100, Italy; 4 Avia-GIS, Risschotlei 33, Zoersel, 2980, Belgium; 5 Fonds National de la Recherche Scientifique, Rue d’Egmont 5, Brussels, B-1000, Belgium

**Keywords:** Spatial ecology, Infectious disease, Remote-sensing, Dynamic model, Longitudinal entomological surveillance network, Mediterranean basin

## Abstract

**Background:**

*Culicoides imicola* KIEFFER, 1913 (Diptera: Ceratopogonidae) is the principal vector of Bluetongue disease in the Mediterranean basin, Africa and Asia. Previous studies have identified a range of eco-climatic variables associated with the distribution of *C. imicola*, and these relationships have been used to predict the large-scale distribution of the vector. However, these studies are not temporally-explicit and can not be used to predict the seasonality in *C. imicola* abundances. Between 2001 and 2006, longitudinal entomological surveillance was carried out throughout Italy, and provided a comprehensive spatio-temporal dataset of *C. imicola* catches in Onderstepoort-type black-light traps, in particular in Sardinia where the species is considered endemic.

**Methods:**

We built a dynamic model that allows describing the effect of eco-climatic indicators on the monthly abundances of *C. imicola* in Sardinia. Model precision and accuracy were evaluated according to the influence of process and observation errors.

**Results:**

A first-order autoregressive cofactor, a digital elevation model and MODIS Land Surface Temperature (LST)/or temperatures acquired from weather stations explained ~77% of the variability encountered in the samplings carried out in 9 sites during 6 years. Incorporating Normalized Difference Vegetation Index (NDVI) or rainfall did not increase the model's predictive capacity. On average, dynamics simulations showed good accuracy (predicted vs. observed r corr = 0.9). Although the model did not always reproduce the absolute levels of monthly abundances peaks, it succeeded in reproducing the seasonality in population level and allowed identifying the periods of low abundances and with no apparent activity. On that basis, we mapped *C. imicola* monthly distribution over the entire Sardinian region.

**Conclusions:**

This study demonstrated prospects for modelling data arising from *Culicoides* longitudinal entomological surveillance. The framework explicitly incorporates the influence of eco-climatic factors on population growth rates and accounts for observation and process errors. Upon validation, such a model could be used to predict monthly population abundances on the basis of environmental conditions, and hence can potentially reduce the amount of entomological surveillance.

## Background

*Culicoides imicola*, one of the major Bluetongue disease (BT) and African horse sickness (AHS) vectors in the old world [1-3] has probably been the most investigated *Culicoides* species over the last decades. A good knowledge of its ecology helps in quantifying potential disease transmission, and assessing the risk of resurgence or expansion of BT/AHS. Therefore, a large number of studies have investigated the spatio-temporal distribution of *C*. *imicola* in the old world [4-10]. Furthermore, since the establishment of BT in southern Europe in 2000 [11], systematic entomological samplings were carried out in many countries in order to monitor the vector populations. An important number of descriptive studies followed [12-19]. Long-term and wide-scale surveys also provided adequate datasets that have supported predicting the vector distribution at large scale as a function of a variety of environmental predictors ([20-32], summed up in Table 1). However, the interpretation of the results across those studies is not straightforward (Table 1). First, because these studies used various modelling approaches, scale and set of environmental predictors. Second, because the correspondence between the timing of the catches and timing of the observation of eco-climatic variables was variable. As a consequence, even if the accuracy of the distribution models was found to be very good, the lack of consistency across study predictions in some regions, and the difficulty in interpreting the relationships that were found, preventing the use of these models for predicting the species distribution outside of the spatio-temporal range of the training data.

**Table 1:** **Summary of the risk studies on *Culicoides imicola *distribution in the Mediterranean basin since 1998**

**References**	**Extent**	**Resolution**	**Dependent variable**	**Statistical model**	**Cofactors selected through the analysis**	**External evaluation**
[21,22]	*Morocco, * *Morocco*/ *Iberia*	*Not given*	*Abundance*	*Linear regression*	*NDVI* (*min*), *windspeed*	*No*.
[23]	*Iberia*	*Size of sampled sites*	*Pres*./*abs*.	*Logistic regression*	*Mean monthly T*° (*min* &*max*), *number of months in the year with mean T*° *exceeding 12*.*5* °*C*	*No*.
[24]	*Iberia,* *Morocco*	*8 km x 8 km*	*Abundance*	*Discriminant analysis*	*8*-*variables model with DEM and Fourier*-*transformed NDVI*, *MIR*, *VPD and LST* (cf. *Table*2)	*No*.
[25]	*Italy,* *Calabria*	*Size of sampled sites*	*Pres*./*abs*.	*Logistic regression*	*T*° (*min* &*max*)	*No*.
[26]	*Portugal*	*1 km x 1 km*	*Both*	*Discriminant analysis*	*DEM and Fourier*-*transformed LST*, *NDVI*, *MIR and TAIR including seasonal cycles*	*No*.
[27]	*Italy*	*10 km x 10 km*	*Pres*./*abs*.	*Multiple logistic regression*	*Mean altitude and mean annual daily min T*° *and relative humidity*	*No*.
[28]	*Sicily*	*1 km x 1 km*	*Pres*./*abs*.	*Stepwise discriminant analysis*	*10*-*variables model with Fourier*-*transformed LST*, *NDVI*, *MIR and TAIR* (cf. *p93*)	*No*.
[29]	*Italy*	*Cell size* = *0*.*0387*°	*Pres*./*abs*.	*Additive model*	*Elevation*, *slope*, *aridity index*, *landuse*, *animal density*, *soil type*, *T*° *and NDVI for the 4 seasons*	*No*.
[30]	*Italy*	*250 m x 250 m*	*Both*	*Discriminant analysis*	*Min*. *T*°, *aridity index*, *altitude*, *slope*, *NDVI and forest cover*	*No*.
[31]	*Spain*	*1 km x 1 km* (*sometimes 8 km x8km*)	*Pres*./*abs*.	*Logistic regression*	*Mean NDVI*, *sun index*, *interpolated precipitations and T*° *and their seasonality*	*Yes*.
[32]	*Spain*	*UTM 10 km x 10 km*	*Abundance*	*GLM neg*. *bin*. &*variation partitioning*	*21*-*variables model with spatial location*, *topo*-*climatic variable*, *domestic and wild hosts*, *soil*, *NDVI and seasonality*	*No*.

DEM, Digital Elevation Model; MIR, Middle Infrared Radiance; VPD, Vapour Pressure Deficit; TAIR, Air temperature. *NB*: A review for some studies that occurred until 2004 is also provided in Baylis et al. [20].

In this study, the set of eco-climatic predictors used to predict *C*. *imicola* abundances was restricted to a few variables measured either by weather stations (temperature, rainfall) or by remote sensing (Land Surface Temperature (LST), Normalized Difference Vegetation Index (NDVI)). In contrast to previous studies, these indices were tested against *C*. *imicola* population using a spatially and temporally explicit model, *i*.*e*. each catch was statistically tested against the eco-climatic conditions that were measured in the matching trapping site, and previous month. The aim of the study was therefore (i) to describe the influence of these eco-climatic indicators on the monthly abundances of *C*. *imicola* measured in Sardinia from 2001 to 2006 and (ii) to predict the observed spatio-temporal dynamics on that basis. The applied perspective of this research consisted in introducing a simple, but yet robust method to analyse data collected through longitudinal networks of entomological surveillance and to map their abundances both in space and time.

## Methods

### Data

**Figure 1:**
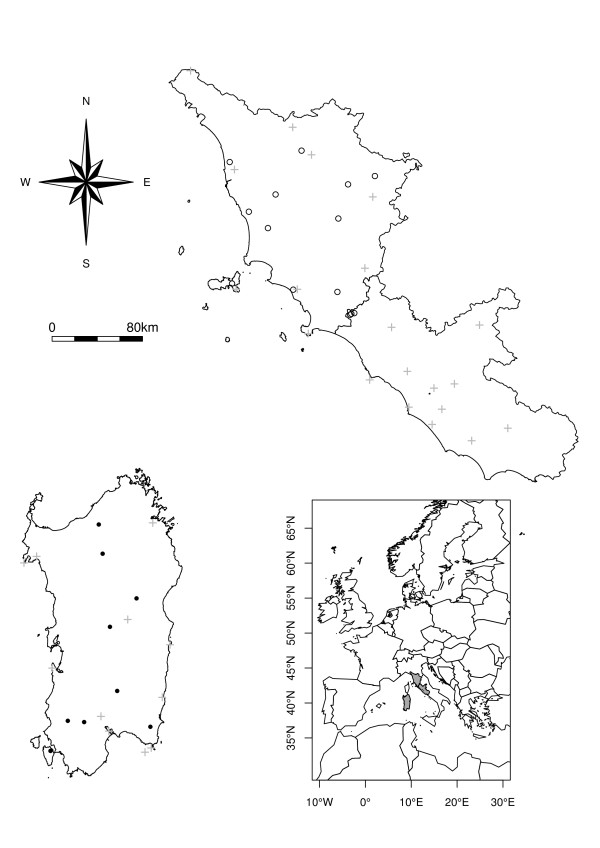
**Study area. ***C*. *imicola* population dynamics were studied on the basis of weekly samplings carried out in Sardinia (bottom left region) at the level of 9 permanent light-traps from 2001 to 2006 (black dots). During the same period, meteorological data were collected at the level of 31 weather stations (grey crosses). Data gathered outside the study area (mainly Tuscany, upper right region on the top) were used to evaluate the model built outside its range of training (open circles).

Between 2001 and 2007, more than 200 permanent Onderstepoort-type black-light traps were operated weekly in Italy in accordance with standardized surveillance procedures of the National Reference Centre for Exotic Diseases [16]. *Culicoides imicola* is not present everywhere in Italy, and can be considered to be endemic in Sardinia region [13,33]. Since the present study aimed to better understand factors affecting *C*. *imicola* seasonality, 9 sites from Sardinia were selected (Figure 1, black dots). In order to filter the high variability in *C*. *imicola* trap catches, data were aggregated for each site by month and maximum abundances were retained, as in previous studies [34]. In order to fill existing gaps, linear regulations implemented in the R library PASTECS [35] were applied, and 49 observations were filled that way. In complement, eleven sites distributed in Lazio (1 site) and Tuscany (10 sites) (Figure 1, open dots) were used to evaluate the extrapolation capacity of a selected set of models.

The model aimed to compare two sets of predictors in their potential to predict *C*. *imicola* seasonality. The first set was made of data collected in weather stations (WS). These data give a good measure of the conditions actually observed on the ground over time, but their relatively limited number does not allow the quantification of spatial variations and heterogeneity. The second set was made of satellite remotely sensed variables. These measures can be affected by various processes taking place between the ground and the sensor, but they have the advantage of better capture of the spatial distribution of the variable of interest, and in doing so, have a good chance to provide an accurate estimate at the locations of the traps.

Meteorological data were obtained from the Italian Air Force Meteorological Service. They were collected between 2000 and 2007 in 105 weather stations distributed across Italy. Eleven of them are found in Sardinia and 21 cover Tuscany and Lazio (Figure 1). Daily observations include temperature (min, max,), rainfall (cumulated value in the first half (12 hours) of the day, cumulated value in the second half (12 hours) of the day), relative humidity (RH), wind direction and intensity. After an aggregation by month, mean temperatures, mean and cumulated rainfalls were used in our analyses. These data were interpolated using ordinary kriging [36]. The empirical semivariogram was modelled using a circular model for temperature and a spherical model for rainfall with initial sill, range and nugget fixed to 8.5, 100 000 and 0.1 respectively. Goodness-of-fit of the semivariogram model was evaluated after leave-one-out cross validation [36]. Output resolution was set as 0.01 decimal degree in accordance with the resolution provided by RS products.

Remotely-sensed eco-climatic variables included the Land Surface Temperature and Normalized Difference Vegetation Index. Day-time and night-time LST products (MODIS Terra MOD11A2) composited at an 8-day interval and the NDVI (MODIS Terra MOD13A2) composited at an 16-day interval were downloaded from the Land Processes Distributed Active Archive Centre. Additionally, the surface reflectance data (MODIS Terra MOD9A1) composited at a 16-day interval were also downloaded. These data were mosaicked and resampled from the original metric sinusoidal projection system to decimal degrees (WGS84) using a nearest-neighbour resampling and with an output resolution of 0.01 decimal degrees. The images were then subjected to a spline interpolation to remove the missing gap [37].

These data were aggregated by month, keeping the minimum, maximum and mean values.

### Model

The discrete-time population model was built in three steps.

The first step consisted in building an autoregressive model where the abundance of populations was predicted using a linear combination of (i) the population in the previous time step (autoregressive term) and (ii) eco-climatic variables measured the month before (equation 1).

(1) $$N_{\text{s,t}}=N\left(\mu_{obs,}\;\sigma_{\mathrm{obs}}\right)$$

(2) $${\text{μ\textasciitilde{}aN}}_{\text{s,t}-1}\text{+b}\;{\text{Var}}_{1}\text{+c}\;{\text{Var}}_{2}{\text{+…+zVar}}_{n}+Intercept$$

where N_s,t_ and N_s,t-1_ are respectively the population sampled in site s at time t and t-1; Var_1_, Var_2_, …, Var_n_ are the eco-climatic predictors; a, b, …, z and *Intercept* are the model parameters.

In the second step, we assumed that the measured population abundances can be quantified from equation 2.

(3) $$N_{t}{\text{=N}}_{t-1}*\exp\left(r*\Delta t\right)$$

After the log-transformation of both sides of equation 2, assuming a constant time interval (in our case a month) between 2 samples and making the relationship spatially explicit (time *t*, site *s*), equation 2 become:

(4) $$LnN_{s,t}=Ln\;N_{s,t-1}+r_{s,\Delta t}$$

One can note that this relationship is very similar to equation 1. Combining the two approaches, we can assume that the linear relationship found between eco-climatic variables and the log-transformed abundances found at time *t* in site *s*, corresponds to growth rate*r* found in equation 3. The right-hand part of equation 1, *i*.*e*. the intercept and additional cofactors differing from the autoregressive cofactor, will therefore be considered as proxies for predicting the monthly increase in population. This approach allows more straightforward biological interpretation of the inclusion of cofactors in the right-side hand of the linear model. The parameters were estimated using a Generalized Linear Model (GLM) and accounting for a normally distributed error term. This model was initially built to estimate model coefficients, *i*.*e*. the relationship that exists between population abundances and co-factors, their range of confidence and the dispersal in residuals.

Finally, we aimed at evaluating how process and observation error could influence the precision and accuracy of the model parameters and predictions [38,39]. Because no prior information on the magnitude of the different sources of observations error in our datasets was available, we evaluated the influence of several levels of observation error ( = 1/2, 1 or 2 times the total variance found in the whole dataset) on the estimates of model parameters, the dependence between population abundances at time *t* and in the previous month (coefficient of the autoregressive cofactor) and the dispersal in residuals ( = process error).

Based on the parameters identified by the GLM statistical model, the space-time dynamics of *C*. *imicola* populations was simulated by seeding 13 individuals at t_0_ (here, March 2001). Population in month t_1_ was predicted by applying the GLM model coefficients to the initial number of individuals seeded and eco-climatic variables measured in the previous month t_0_. Population at time 1 was then used to predict population at time 2, and the predictions were iterated through the time series until December 2006. At each time step, both process and observation error values were added to the predicted abundance of population. These value were both sampled from a normal distribution with a zero-mean and a standard deviation equal to that found in the model residuals for the process error, and equal to that of the overall log-transformed catches for the observation error. The seed value of 13 individuals was chosen because it was the mean abundance encountered in the months of March from 2001 to 2006 in Sardinia.The predictions were repeated over 100 simulations, and averaged.

Finally, using a similar approach with a seed of 2 individuals in March 2005, simulations were carried out over the entire spatial domain of Sardinia (extent: 8 to 10°E; 38.8 to 41.4°N) until December 2006. The approach allowed production of monthly maps of *C*. *imicola* relative abundance at a spatial resolution of 0.01 decimal degree. The maps presented in the results section were produced on the basis of 250 bootstraps. For each of the bootstraps, the coefficients of all terms (constant, autoregressive co-factor and covariates) were sampled from a normal distribution of coefficients with mean and standard deviation estimated by the GLM model. The GLM model was finally applied to Tuscany to test its predictions against the observed catches.

All analyses were implemented under R [35], using the packages MASS [40], raster [41], rgdal, PASTECS and gstat [36].

## Results

The GLM coefficients used in the spatio-temporal dynamic model are presented in Table 2. Most of the explained variability predicted by the GLM resulted from the inclusion of an autoregressive term (Table 2; multiple r-squared r2 = 0.65, p < 0.001), then from temperature (LST, r2 = 0.25, p < 0.001; or T from WS, r2 = 0.36, p < 0.001). When combined, the autoregressive cofactor and LST or WS temperature predicted respectively 75.6% or 75.9% of the total variability. Other co-variables such as altitude (r2 = 0.16, p < 0.001), rainfall (r2 = 0.01, p < 0.01) or NDVI (r2 = 0.25, p < 0.001; r2 = 0.70, when tested with the autoregressive cofactor) had a much lower predictive power. The correlation coefficients between observations and GLM predictions based on remote-sensing or weather stations data were very similar (r corr = 0.87 and 0.88, respectively). Predictions made in Tuscany resulted in overestimating the populations in most sites where it was applied (Additional file 1: Figure S1).

**Table 2:** **Coefficient estimates** (**with 95% CI**) **from GLM carried out with RS or WS data respectively**

**Coefficients**	**Estimates**	**Pr(>|t|)**	**95%CI**		**AIC**	**Multiple R2**
RS						
Intercept	−1.73	***	−2.62	−0.85	2165.7	0.766
Autoreg	0.70	***	0.65	0.74		
LST	2.25	***	0.18	0.32		
LST2	−0.0038	***	−0.0058	−0.0018		
NDVI	−0.13	ns	−1.22	0.95		
Altitude	−0.0011	**	−0.0018	−0.00044		
WS						
Intercept	−1.66	***	−2.42	−0.90	2160.2	0.768
Autoreg	0.64	***	0.59	0.68		
T	0.22	***	0.12	0.31		
T2	−0.0016	ns	−0.0047	0.0012		
Rainfall	0.046	p = 0.06	−0.0023	0.095		
Altitude	−0.0015	***	−0.00219	−0.00084		

**Figure 2:**
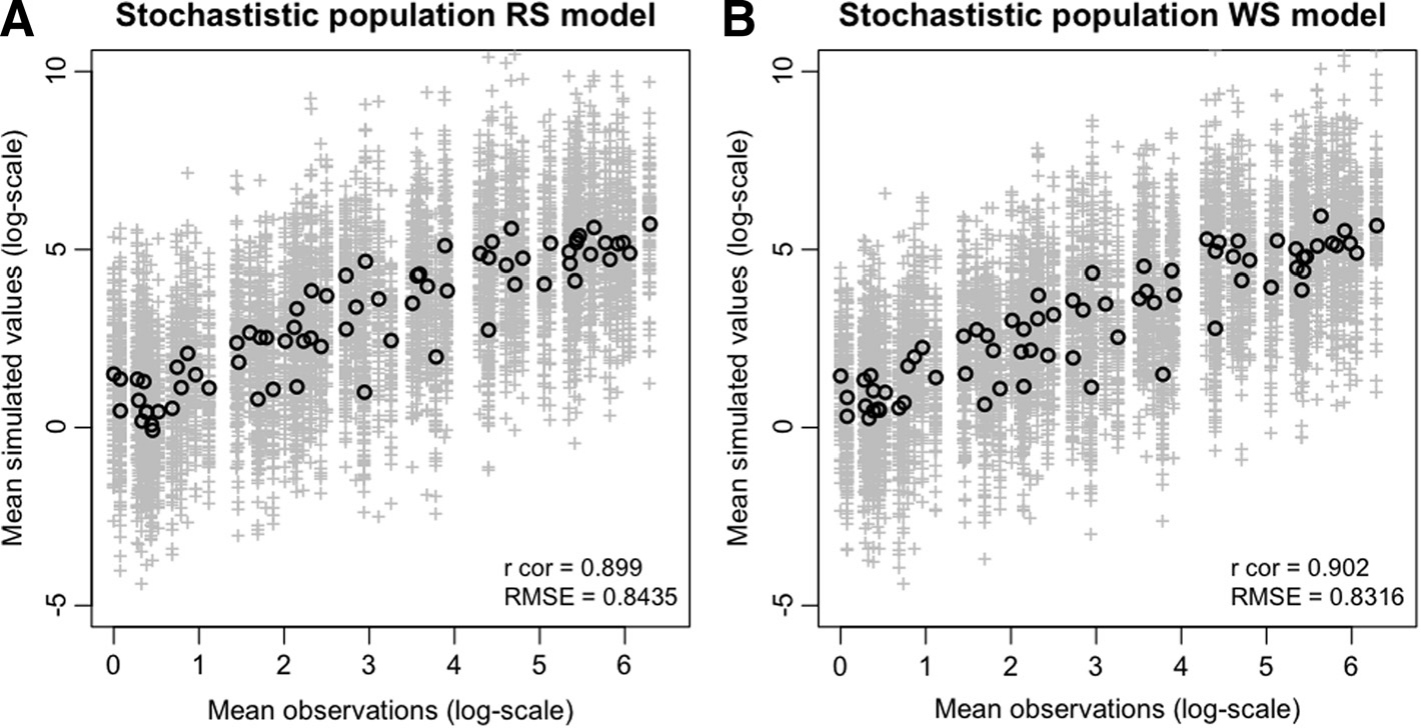
**Evaluation of the dynamic models' accuracy and precision.** Comparisons among observed *C*. *imicola* log-abundances and the predictions carried out with the population dynamic model (99 simulations in grey and the average is given by black dots), using RS data (**A**) or WS data (**B**).

**Figure 3:**
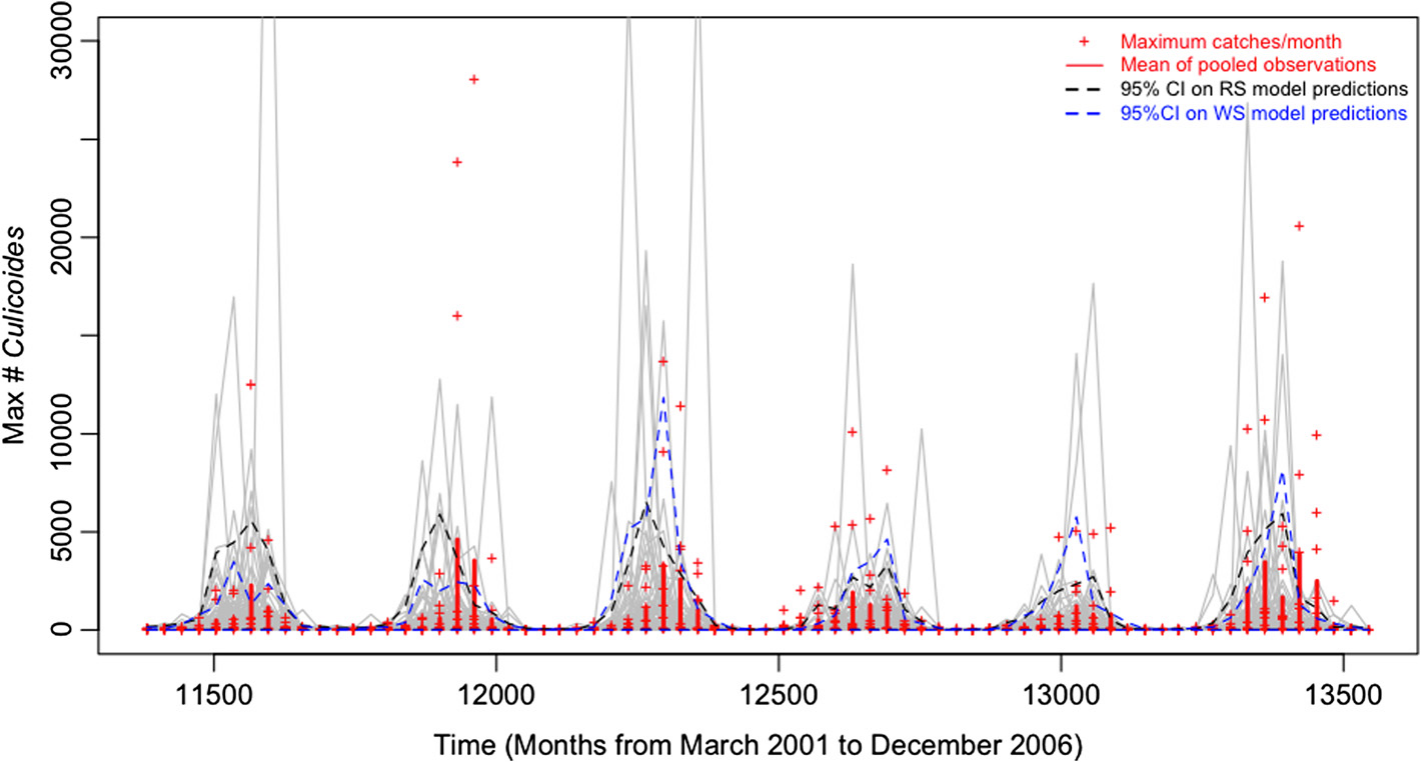
**Seasonal activity of *C. imicola *during 6 years (9 sites).** Grey lines present the result of 99 stochastic simulations with RS data and accounting for process and observational errors. 95% confidence intervals are presented in black (RS data model) and in blue (WS data model). Red lines and dots represent respectively mean monthly abundances found in all sites grouped and monthly maximal abundances for each sites.

The main results of the simulation models are presented in Figure 2. Simulations had lower predictive power than the GLM’s (Table 3) and were not able to reproduce the level of all peaks of measured populations (Figures 2 and 3). Nevertheless, they succeeded in reproducing the seasonality of the populations (Figure 3). The predictions of the model also allowed reproducing the marked difference between *C*. *imicola* catches made at different sites and the extinction that could be observed in high elevation sites more specifically in Austis, for example (Additional file 2: Figure S2 and Additional file 3: Figure S3).

**Table 3:** Goodness of fit found for the GLM and the dynamic model run with RS or WS data

	**Training model**		**Dynamic model** (**99 stochastic realizations**)			
			***Whole extent (site level)***		***Mean model (regional level)***	
*Data*	*RS*	*WS*	*RS*	*WS*	*RS*	*WS*
Rcorr	0.88	0.88	0.71	0.75	0.9	0.9
RMSE	1.361	1.355	1.97	1.86	0.84	0.83

Indicators of goodness of fits for the dynamic model are also presented on Figure 2.

**Figure 4:**
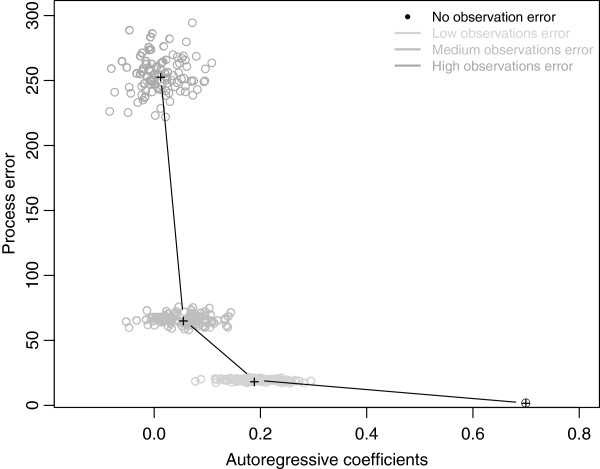
**The effect of unknown observational error on process error estimates, variance and the autoregressive cofactor.**

Increasing the observational error increased the lack of precision on the estimate of model coefficients (Additionial file 4: Figure S4) and on process error (Figure 4). In particular, the average estimate of the autoregressive cofactor coefficient was most influenced by observation error, and tended towards zero when observation error increases (Additionial file 4: Figure S4). As expected, increasing observational error also affected process error estimates and the link that exists between process error and the autoregressive term (Figure 4).

**Figure 5:**
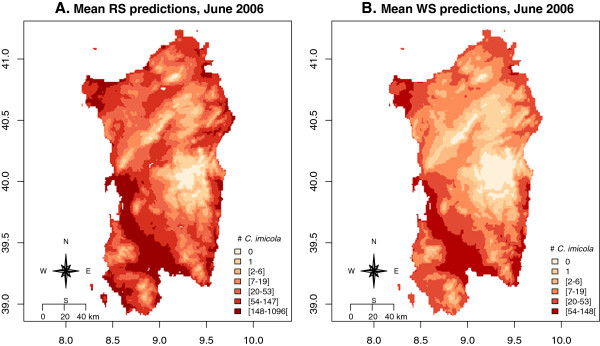
**Distribution maps of the relative mean abundances of *C. imicola *found for June 2006.** The mean abundances presented on these maps were realized after 250 bootstraps of the dynamic model that account respectively for RS data (**A**) and WS data (**B**). These simulations were realized after seeding 2 individuals in each pixel in March in 2005. A mask for altitudes lower than 0 meter was applied.

We present two distribution maps of *C*. *imicola* predicted abundances for the month of June 2006 (Figure 5). There are no apparent important differences in terms of relative abundances between the predictions made with RS data in comparison to those built with the WS data. Both highlighted that abundances are low at high altitudes, and also allowed differentiation of the different abundance classes within areas of constant elevation. Finally, a comparison of predicted distribution over time highlights the months when the highest activity occurred and the specific areas where this highest activity could happen.

## Discussion

As expected (e.g. see [6], but also Table 1), temperature -and its quadratic value (for LST)- influenced *C*. *imicola* population abundance. Veronesi *et al*. showed experimentally that temperature could influence the duration and survival of sub-adult stages in *C*. imicola [42]. In our models, temperature from WS, when tested as a single predictor, explained 36% of the variability measured in the dataset investigated, which is ~10% higher than MODIS LST variance explanation. This range (from 27 to 36%) is lower than the variability in *C*. *imicola* catches explained on the basis of WS temperature and LST in previous studies, where values ranging between 34 to 40% were found [9]. Combining temperature with a first-order autoregressive cofactor increased significantly the explanatory power of the model.

Whilst the strong effects of temperature and autoregressive cofactors were somewhat anticipated, we were surprised to find that including NDVI did not substantially improve the predictive power of the model. NDVI could indeed be believed to improve spatial predictions by highlighting areas where moisture conditions are more favourable to *C*. *imicola* larval stages [43-46]. In addition, it was previously a variable improving *C*. *imicola* distribution models [20]. One possible explanation is the role played by artificial breeding sites in the direct vicinity of the traps. Indeed, all traps are placed in farms where a variety of artificial breeding sites can be found: mud surrounding local provision of livestock drinking water, local small streams of cleaning water, or even small-scale irrigated pastures. All these could provide *C*. *imicola* populations suitable habitats surrounding the trap, even in the absence of surrounding vegetation that could be detected at larger-scale by the remotely sensed NDVI signal. In addition, compared to other studies, NDVI may not be such a limiting factor in Sardinia where catches appear to be very abundant over the entire region. A similar approach developed at the fringe of the *C*. *imicola* distribution range may hence highlight a relatively stronger contribution of factors other than temperature in modelling *C*. *imicola* population dynamics. The fact that the model overestimated the populations in Tuscany suggests that the model would need further adjustments to account for a different range or value in the environmental conditions than those encountered in Sardinia alone.

It is obvious that the model simulations had lower predictive power than the initial statistical approach built to find estimates of cofactors (lower correlation coefficient and higher RMSE, Table 3). Indeed the reduction in predictive power can be explained by the fact that the simulations only use a constant initial population at t_0_ (here 13 individuals in March 2001) to feed the predictions over the entire time series whereas the predictions of the statistical model are estimated with the observed abundances measured at each previous time step. In other words, predictions from the model simulations are not made based on the observed population at the previous time step, but are based on the modelled population at the previous time step, hence the reduction in predictability. However, the comparative advantage is that the simulation demonstrated moderate to good predictability over space and time simply based on the spatio-temporal distribution of the predictors, and do not require field samplings of *C*. *imicola* to make the predictions.

Those simulations succeeded fairly well in reproducing the seasonality of the populations, the maintenance of *C*. *imicola* activity during winter, even at very low population levels, and the likely outcome of extinction at high elevation ( > 500 m). Even if the simulations were not able to fully quantify the level of the peaks of maximum abundances, they described very well the increase in population activity that occurs at the beginning of each season (Figure 3). The applied perspective of such a characteristic could be found in the development of a surveillance system that could predict seasonal vector abundances on the basis of the current temperature and could help to alert on periods of high risk of bluetongue disease transmission. The model predicted extinctions at high elevation (Additional file 2: Figure S2 and Additional file 3: Figure S3), and the maintenance of the activity of vectors in these areas would require renewed introductions. A further development of the model could account for external introduction of novel specimens. For example, it could be coupled to broad-scale wind density models such as presented in [47,48], transport and trade networks [49], or to local-scale leptokurtic models, which describe the decrease in *Culicoides* spp. abundances as a function of the distance to the farm [50]. Other factors, not accounted for in this study, are likely to influence *C*. *imicola* populations. One such factor may be the local density of livestock (horses, cattle, sheep and goats), which provides both hosts for blood feeding females, and breeding sites through the manure. Sardinia hosts approximately 3.9 million sheep and goats, the highest density in all of Italy. Although this number does not show strong seasonal fluctuation, grazing patterns are strongly seasonal in the most elevated part of the island, with sheep flocks free-grazing in the pastures. In contrast, most sheep are grazing in pastures in the direct vicinity of farms in the low-elevation parts of Sardinia. These factors may also influence the spatio-temporal pattern of *C*. *imicola* populations, but quantification of these effects is difficult due to a lack of high-resolution data on hosts and grazing patterns. In addition, Onderstepoort-type black-light traps catches do not accurately reflect host-seeking behaviour by biting midges in comparison to host-baited traps catches [51], hence limiting the use of our dataset to test for the local effect of hosts distribution on *C*. *imicola* populations abundance.

Average correlation coefficients between observations and predictions were very similar between the RS model and the WS model (Figure 2). The model with RS predictors has nevertheless the advantage that predictions can be made over all pixels without interpolation of observations such as is needed in the case of weather station data. Furthermore, interpolation tends to produce very continuous surface that do not fully reflect the local heterogeneities in temperatures (Figure 5A and B). This comparative advantage could not be quantified in our study, probably because the observation error in catches could be higher than the difference in predictions due to the differences in the type of temperature data. Another possible explanation is that local temperature at the level of the trap, influenced by local conditions (shelter and shading, local topography) could be as different from the RS data as it is from the WS data. This could only be evaluated thoroughly using local temperature measurements with data loggers. Overall, the distribution that we predicted at the seasonal peak is fairly similar to that observed by previous studies, (e.g. [13,33]), with low-level population areas located at high altitudes.

Our modelling approach included at least three sources of uncertainties. The first one appears when an autoregressive cofactor is included and results from the interplay between process and observation error [38]. We tried to take it into account by (1) quantifying the effect of observation error on coefficient estimates, (2) trying to quantify the influence of observation error on process error, and (3) including using a stochastic component in the modelling framework.

As expected, introducing different levels of observation error influenced both GLM coefficient estimates [39], and the extent of process error (Figure 4). Since we have little prior information on the magnitude of observational error, we decided to run our simulations with a fairly high level of observational error, introduced in the form of an error term sampled from a normal distribution with 0-mean and standard deviation equal to half the variance found in the whole dataset. This introduces a lot of variability in the predictions, and one way to reduce uncertainties could only be gained by experimental quantification of observation error. Other methods to include explicitly observation and process error are available (e.g. state-space models such as in [52] or else, [38] and [39] for implementations using the Bayesian techniques; mixed models, such as highlighted in [53]; hybrid models developed in [54]). Our approach was somewhat simpler, but allows quantification of the impact of observation error to be made under various modelling frameworks, such as for examples BRT [55], GLMM [56] or autologistic models [57].

## Conclusion

Spatially and temporally explicit models have considerable prospects for modelling data arising from longitudinal entomological surveillance because they allow the incorporation of seasonality explicitly in the model and facilitate interpretation of the results by identifying eco-climatic factors that influence population growth rate in space and time (see also [58]). Once validated, these models could be used to predict population levels on the basis of observed environmental conditions, hence potentially reduce the amount of entomological surveillance. Together with the recent advances in methods for the identification of biting midges [59,60] and blood meal sources [61,62], these models should help to strengthen ecological studies on biting midges, a field by far underexplored. A further improvement of these models would be gained by a better quantification and integration of observation error.

## Competing interests

The authors declare that they have no competing interests.

## Authors’ contributions

TR, AC, MGo, ED, GH and MG conceived the study in the framework of the EPISTIS project. AC & MGo performed samplings, identification and global management of the entomological material; ED & GH extracted and processed RS data. TR & MG performed the analyses. TR wrote the first draft of the manuscript. All the authors contributed to the writing and approved the final version of the manuscript.

## Supplementary Material

Additional file 1 **External evaluation of the statistical model on 10 sites located in Tuscany and 1 site in Lazio (see Figure** 1**).** Click here for file

Additional file 2 **Seasonal activity of *C. imicola *observed at the level of the 9 sites considered in the study (dots) and predicted by the dynamic model using RS data.** Areas in grey came from 99 simulations accounting for observation errors; black lines show average model predictions and dotted lines correspond to the 95% CI. Data are presented in log-scale. Click here for file

Additional file 3 **Seasonal activity of *C. imicola *observed at the level of the 9 sites considered in the study (dots) and predicted by the dynamic model using WS data.** Click here for file

Additional file 4 **The effect of unknown observational error on coefficient estimates. Four arbitrary levels of observational errors were selected.** Plots resulted from 99 bootstraps Click here for file
