# Supplementary figures and images for: Predicting the spatio-temporal distribution of Culicoides imicola in Sardinia using a discrete-time population model
Source: Parasit Vectors. 2012 Nov 22;5:270. doi: 10.1186/1756-3305-5-270 (PMC3561275; doi:10.1186/1756-3305-5-270)

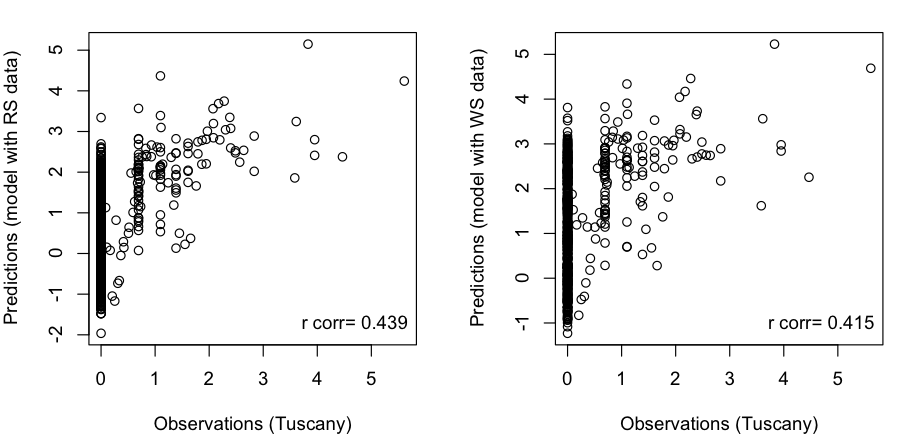

Supplement: Additional file 1 — External evaluation of the statistical model on 10 sites located in Tuscany and 1 site in Lazio (see Figure 1). [file 1756-3305-5-270-S1.png]

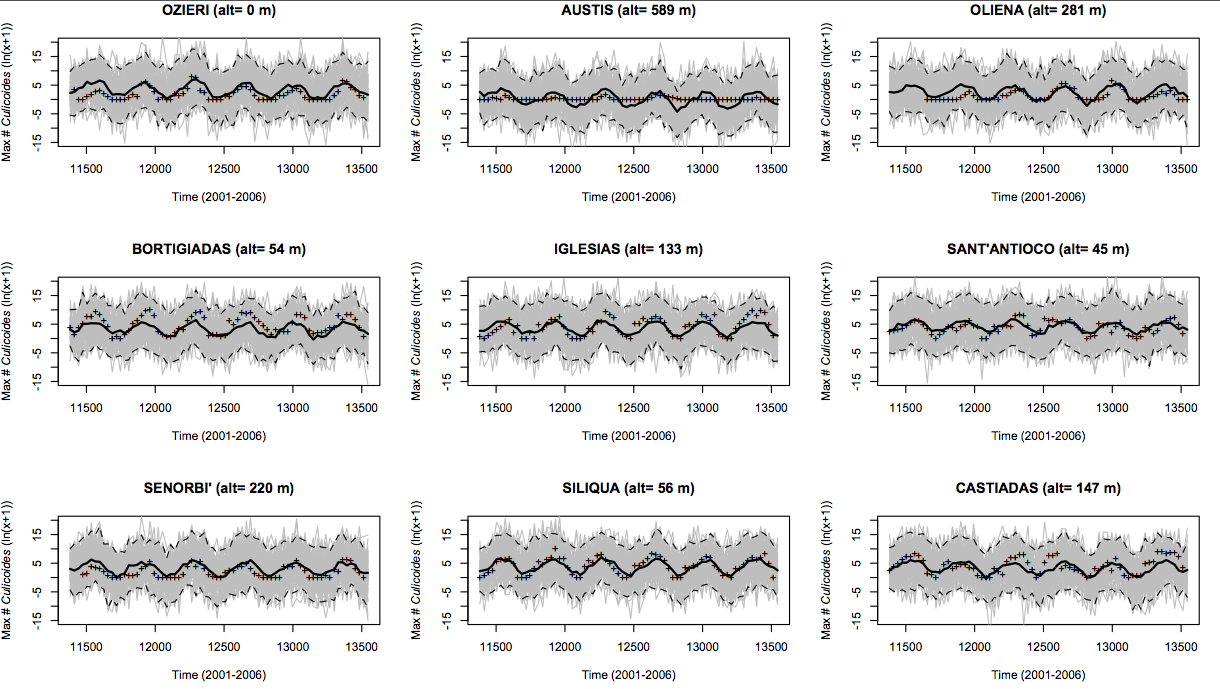

Supplement: Additional file 2 — Seasonal activity of C. imicola observed at the level of the 9 sites considered in the study (dots) and predicted by the dynamic model using RS data. Areas in grey came from 99 simulations accounting for observation errors; black lines show average model predictions and dotted lines correspond to the 95% CI. Data are presented in log-scale. [file 1756-3305-5-270-S2.png]

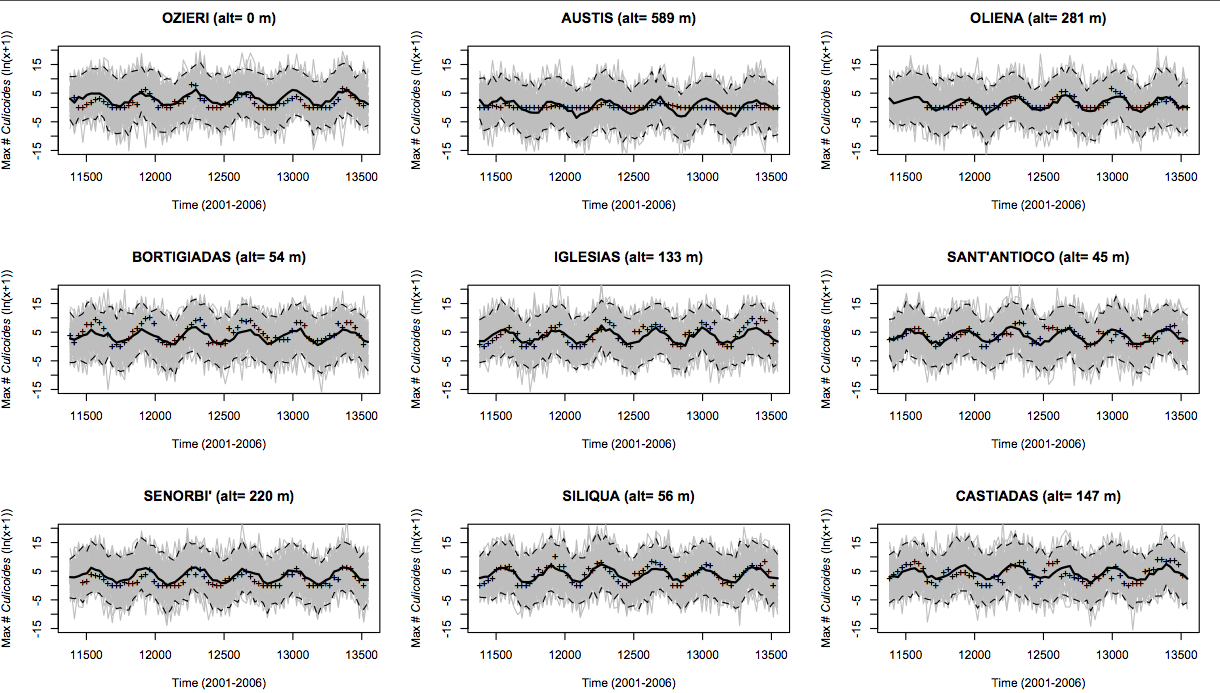

Supplement: Additional file 3 — Seasonal activity of C. imicola observed at the level of the 9 sites considered in the study (dots) and predicted by the dynamic model using WS data. [file 1756-3305-5-270-S3.png]

**(Intercept)**

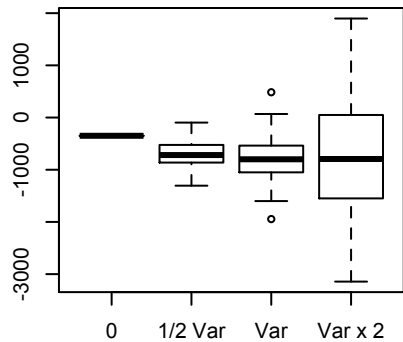

**LogLag**

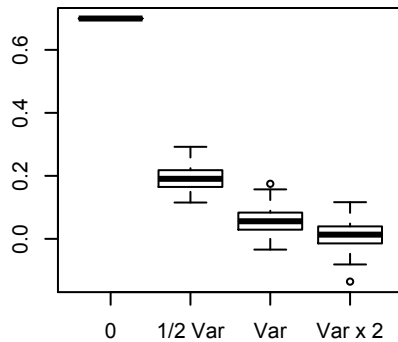

**MeanLST**

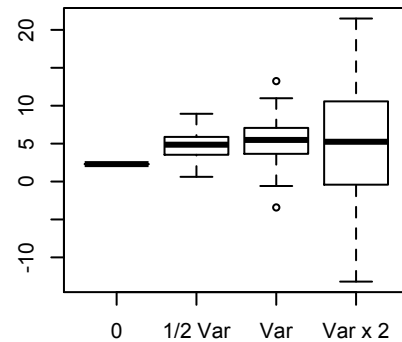

**I(MeanLST^2)**

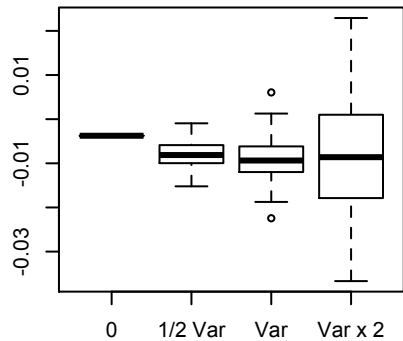

**MeanNDVI**

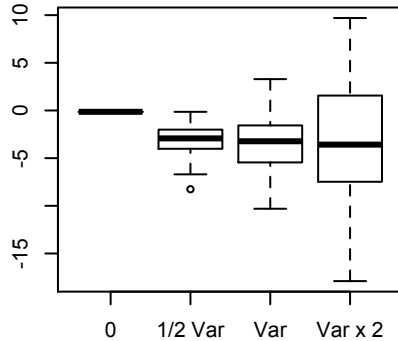

**Altitude**

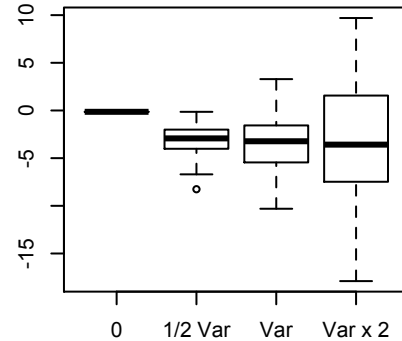

Supplement: Additional file 4 — The effect of unknown observational error on coefficient estimates. Four arbitrary levels of observational errors were selected. Plots resulted from 99 bootstraps [file 1756-3305-5-270-S4.pdf]
